# Life‐History Responses of the Fall Webworm *Hyphantria cunea* to Temperature Change: Not Following the Temperature–Size Rule

**DOI:** 10.1002/ece3.72225

**Published:** 2025-10-01

**Authors:** Hua Lu, Li‐Li Huang, Liang Chen, Sheng‐Bin Wu, Fang‐Sen Xue, Xing‐Ping Liu, Hai‐Min He

**Affiliations:** ^1^ College of Forest Jiangxi Agricultural University Nanchang China; ^2^ College of Ecology and Environment YuZhang Normal University Nanchang China; ^3^ Hubei Forestry Pest Control and Quarantine General Station Wuhan China; ^4^ Wuhan Lvyi Chutian Ecological Technology Co., Ltd Wuhan China; ^5^ Institute of Entomology Jiangxi Agricultural University Nanchang China

**Keywords:** body weight, development time, growth rate, *Hyphantria cunea*, sexual size dimorphism, temperature

## Abstract

Temperature serves as a critical environmental factor for ectotherms and significantly impacts numerous life history traits. This study examined the influence of temperature on life‐history traits of the fall webworm *Hyphantria cunea* at 20°C, 22°C, 24°C, 26°C, and 28°C. There were no significant differences in larval survival rates across the temperatures tested. However, pupal survival at 28°C was significantly lower compared to other temperatures. Sex ratios remained consistent at 20°C–26°C but skewed toward males at 28°C. The developmental time from egg to adult decreased as the temperature rose, with males emerging earlier than females (protandry), primarily due to shorter larval stages. Pupal weight exhibited temperature‐ and sex‐dependent trends: female pupae peaked at 22°C (184.6 mg) and there was a 9.2% increase at 28°C as compared with 20°C, demonstrating a reverse temperature‐size rule. In contrast, male pupal weight decreased by 8.2% at 28°C, indicating greater thermal sensitivity. Growth rates increased with temperature, showing sex‐specific variations at lower temperatures. Adult weight mirrored pupal trends, with females consistently larger than males. Metamorphic weight loss increased with temperature in both sexes, but males experienced more pronounced losses. Female‐biased sexual size dimorphism (SSD) is inclined to increase along with weight gain and temperature, not conforming to the Rensch's rule. Furthermore, positive relationships were discovered between pupal weight and larval development time, and between adult weight and fecundity. These findings emphasize temperature‐driven plasticity in development, SSD, and thermal tolerance, highlighting the species' adaptability to warming climates and its implications for pest management strategies.

## Graphical abstract

The life‐history traits of the fall webworm were examined at 20°C, 22°C, 24°C, 26°C, and 28°C. Larval development time was shorter in males than females at all tested temperatures. Female body weight increased at high temperatures, while male body weight decreased. Weight loss and the sexual size dimorphism both increased with rising temperature. Female pupal weight exhibited a significant positive correlation with fecundity.

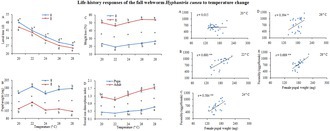

## 1 | Introduction

The majority of insects are ectothermic (cold‐blooded), meaning their body temperature fluctuates in direct correlation with environmental conditions. Consequently, temperature serves as the primary driver of growth and development when food supply is not a limiting factor. Within an optimal temperature range, an increase in temperature will accelerate insect metabolism, thereby enhancing developmental rates. Each species and life stage may exhibit distinct developmental responses to temperature variations. Generally accepted guidelines indicate that increased temperatures can result in higher growth rates, shorter developmental periods, and smaller adult size in insects and other ectotherms (Sibly and Atkinson 1994). This phenomenon, referred to as the ‘temperature‐size rule’ (TSR), has been witnessed in more than 80% of the studied ectothermic species, covering various organisms like animals, plants, protozoa, and bacteria (Atkinson 1994). However, exceptions exist, including well‐recorded cases of the reverse TSR, in which mature body sizes increase at higher temperatures (Atkinson 1995; Willott and Hassall 1998; Kingsolver et al. 2007). Notably, many of these exceptions pertain to insects across various orders, including Lepidoptera (Atkinson 1994). Among the 67 insect studies summarized by Atkinson (1994), 75% indicated significant reductions in size as temperature increased (adhering to the TSR), while 18% revealed significant increases in size, and 7% showed maximal size at intermediate temperatures. Significant increases in size along with temperature have been witnessed in diverse insect species. For instance, this phenomenon has been noted in mayfly species (Atkinson 1995), British grasshopper species (Willott and Hassall 1998), the tropical butterfly, *Bicyclus anynana* (Fischer et al. 2004), the small cabbage white butterfly, *Pieris rapae* (Kingsolver et al. 2007), the rice stem borer, *Chilo suppressalis* (Fu et al. 2016; Huang et al. 2018), the Asian corn borer, *Ostrinia furnacalis* (Xiao et al. 2016; Xia et al. 2019), and the fall armyworm *Spodoptera frugiperda* (Huang et al. 2021). Additionally, maximal size at intermediate temperatures has been reported in the aphid *Acyrthosiphon pisum* (Lamb 1988) and in Drosophila (David et al. 1997). It is clearly established that in ectotherms, both somatic growth and ontogenetic development rates rise along with increasing temperatures, resulting in a shorter time to maturity. Whether the body size at maturity is smaller or larger is determined by the relative temperature sensitivity of these two processes: the TSR takes place when the development rate is more temperature‐sensitive, while the reverse‐TSR is witnessed when the biomass accumulation rate is more sensitive (Zuo et al. 2012). The underlying reasons for which the majority of ectotherms follow the TSR, while only a minority exhibits the reverse‐TSR, have yet to be fully clarified.

The fall webworm, *Hyphantria cunea* (Drury) (Lepidoptera: Arctiinae), is a moth species native to North America and has turned into an invasive species in numerous regions of Europe and Asia since 1940 (Warren and Tadic 1970). It was first recorded in Dandong city, Liaoning Province, China in 1979. Subsequently, the distribution range of the moth has expanded from 31°N to 43.9°N (approximately 1000 km) and from 113.5°E to 126.2°E (approximately 1400 km) (Cao et al. 2016). In August 2016, it was observed for the first time in Dawu County, Hubei Province (31°42′N, 114°33′E), where it became a significant defoliator of deciduous trees (Min et al. 2018). Adults of *H. cunea* possess degenerated mouthparts and are unable to feed (Gomi 2000), making larval weight gain crucial for their lifetime fitness (Jervis et al. 2005). Larvae exhibit a typical aggregation behavior before the fourth instar, continuously spinning silk to encase damaged leaves with silk webs. Gregarious larvae commonly create large nests on the outer branches of deciduous trees to be protected (Ito 1977) and for thermoregulation (Morris and Fulton 1970; Rehnberg 2002, 2006). Webworms spend their larval stage inside these webs, feeding nocturnally and digesting food during inactive diurnal periods (Rehnberg 2006). Rehnberg (2006) reported that web internal temperature frequently exceeds 50°C, with larvae tolerating body temperatures between 40°C and 50°C. The fall webworm undergoes pupal diapause primarily induced by photoperiod; early larval instars are particularly sensitive to this environmental factor (Masaki 1977; Gomi 1997; Chen et al. 2014). Overwintering pupae occur in bark cracks, ground litter, or topsoil (Min et al. 2018). In Dawu County, the moth completes three generations annually, with third‐generation full‐growth larvae descending from trees in late September to seek overwintering sites.

Life‐history traits of insects, including development time, body size, fecundity, longevity, and sexual size dimorphism (SSD), are critical determinants of fitness (Nylin and Gotthard 1998). Pest outbreaks are closely associated with these traits (Fu et al. 2016). This study conducted a systematic examination of the life‐history traits of the southernmost Dawu population of *H. cunea* at various temperatures. Such information is of great value for modeling the predicted spread of the species and formulating effective control strategies.

## 2 Materials and Methods

### 2.1 Insect Material and Culture

On May 31, 2024, more than 500 s‐instar larvae from the first generation of *H. cunea* were obtained from five *Platanus acerifolia* trees in Dawu County, Hubei Province (31°42′N, 114°33′E). These larvae were transferred to 10 transparent plastic boxes (size: 19 × 12.5 × 7 cm) containing fresh *P. acerifolia* leaves and maintained at 28°C with a photoperiod of L:D 15:9 h until the emergence of adults. Each box was inspected daily and replenished with fresh leaves as required. We recorded newly emerged adults daily and definitively determined their sex. Male moths are characterized by pectinate antennae and usually have smaller abdomens, while female moths are characterized by filamentous antennae (Loewy et al. 2013). Newly emerged adults within 24 h were randomly paired and placed into clear plastic cups (8.5 cm high, 6.5 cm diameter) lined with moistened cotton balls for oviposition. Egg masses were collected on a daily basis and transferred to Petri dishes lined with wet filter papers. Eggs were monitored each day until hatching. Neonates derived from these eggs were utilized for subsequent experiments.

### 2.2 | Experimental Design and Measurement Approaches

Once hatching, the larvae were moved to 60 transparent plastic boxes (dimensions: 19 × 12.5 × 7 cm). Each box contained 50 larvae derived from the same egg mass, which fed on the leaves of *P. acerifolia*. For each temperature treatment, the boxes were randomly assigned into six groups (two boxes per group). These boxes were placed in five artificial weather educators boxes (QHX‐300BSH‐III, Shanghai CIMO Medical Instrument Manufacturing Co. LTD, Shanghai, China) set at constant temperatures of 20°C, 22°C, 24°C, 26°C, and 28°C± 1°C, and with a photoperiod of LD 15:9 (15 h light: 9 h dark). When reaching the third instar, only 35 larvae remained in each box, and excess larvae were moved out. The sample size was determined as 420 (35 × 6 × 2 = 420) for each temperature treatment. The survival rate is shown in Table 1. After the larvae reached the prepupal stage, each of them was placed in a Petri dish (height 2.0 cm; diameter 9.0 cm) with wet filter papers for pupation and emergence.

**TABLE 1:** Larval and pupal survival rates (mean ± SE.) and sex ratio of *Hyphantria cunea* at 20°C, 22°C, 24°C, 26°C, and 28°C.

Temperature (°C)	Larval survival rate (%)	Pupal survival rate (%)	Sex ratio (♀: ♂)
20	89.76 ± 2.35 a (*N* = 420)	93.93 ± 1.14 a (*N* = 377)	0.51: 0.49^ns^
22	83.33 ± 2.17 a (*N* = 420)	94.75 ± 1.10 a (*N* = 350)	0.52: 0.48^ns^
24	83.81 ± 3.17 a (*N* = 420)	95.46 ± 0.70 a (*N* = 352)	0.51: 0.49^ns^
26	88.10 ± 5.54 a (*N* = 420)	92.19 ± 1.47 ab (*N* = 370)	0.49: 0.51^ns^
28	82.86 ± 3.02 a (*N* = 420)	89.76 ± 1.88 b (*N* = 348)	0.44: 0.56*

*Note:* Values (±SE) within one column followed by distinct letters are significantly different at the 0.05 level based on one‐way ANOVA and Tukey's HSD multiple tests.

Abbreviation: ns, no significant difference.

* Significant difference of sex ratio from 0.50: 0.50 at *p* < 0.05 level.

For the investigation of egg development under varying temperature conditions, eggs collected within 1 h post‐oviposition were transferred to Petri dishes lined with moistened filter paper and subsequently maintained in five artificial weather educates boxes. Each temperature condition had 3 replicates, with 100 eggs per replicate.

The following traits were documented for all individuals: developmental times for egg, larval, and pupal stages; pupal and adult weight. The survival rates of larvae and pupae were monitored on a daily basis across all temperature conditions. Pupae were weighed on the day after pupation, while adult weights were noted after meconium release. Weighing was performed using an electronic balance (AUY120, SHIMADZU Corporation, Japan). The growth rate was computed as ln (pupal weight)/larval development time (Gotthard et al. 1994). Weight loss between pupation and adult eclosion was computed using the formula: proportional weight lost = 1−(adult weight/pupal weight). The SSD was estimated using a sexual dimorphism index (Lovich and Gibbons 1992), defined as SSD = (size of the larger sex/size of the smaller sex)–1. A total of 1618 individuals (801 females and 817 males) from five temperatures were reared to adulthood and weighed.

After emergence, newly emerged adults within 24 h from five different temperatures were randomly paired and placed into clear plastic cups lined with moistened cotton balls for oviposition. For each temperature, 30 pairs of moths were used. Adults were kept in a rearing room at 26°C± 2°C, 70% ± 15% RH, and under natural photoperiods. Egg masses were collected on a daily basis, and the number of eggs was counted. Clear plastic cups were replaced every day until the adult passed away. Adult developmental metrics, such as mating success, preoviposition and oviposition periods, fecundity, and adult longevity, were recorded.

### 2.3 Statistical Analyses

The statistical software SPSS (version 22.0; IBM, www.ibm.com) was employed for data analysis. The Kolmogorov–Smirnov test was utilized to verify the assumption of normal distribution. A linear mixed model was employed to analyze the effects of temperature, sex, and their interaction on life history traits, taking temperature and sex as fixed main effects and considering the feeding group as a random effect. Tukey's post hoc test was conducted to evaluate differences in life history traits across temperature levels. In order to satisfy the assumptions of normality and homoscedasticity, an arcsine square root transformation was performed on proportional data that approached boundary values (0 or 1). One‐way ANOVA and Tukey's test were employed to compare larval and pupal survival rates, egg time, and biological parameters of mated females across different temperatures. Nonparametric tests, followed by binomial distribution tests, were utilized to calculate sex ratios. Independent samples *t*‐tests were conducted to evaluate sexual size dimorphism between pupae and adults. Linear regression analyses were performed to explore the correlations between larval development time and pupal weight, and also between female pupal weight and fecundity.

## 3 Results

### 3.1 Survival Rate as Well as Sex Ratio

The Larval survival rates did not exhibit significant differences at different temperatures (Table 1, *F*_4,25_ = 1.492, *p* = 0.254). In contrast, pupal survival rates showed significant variations among different temperatures, with the survival rates at 28°C being significantly lower compared to those at 20°C, 22°C, 24°C, and 26°C (Table 1, *F*_4,25_ = 3.013, *p* = 0.037). No significant differences in sex ratio were detected at 20°C, 22°C, 24°C, and 26°C; however, the proportion of males was significantly higher than that of females at 28°C (Table 1, *p* < 0.05).

### 3.2 Development Time

**FIGURE 1:**
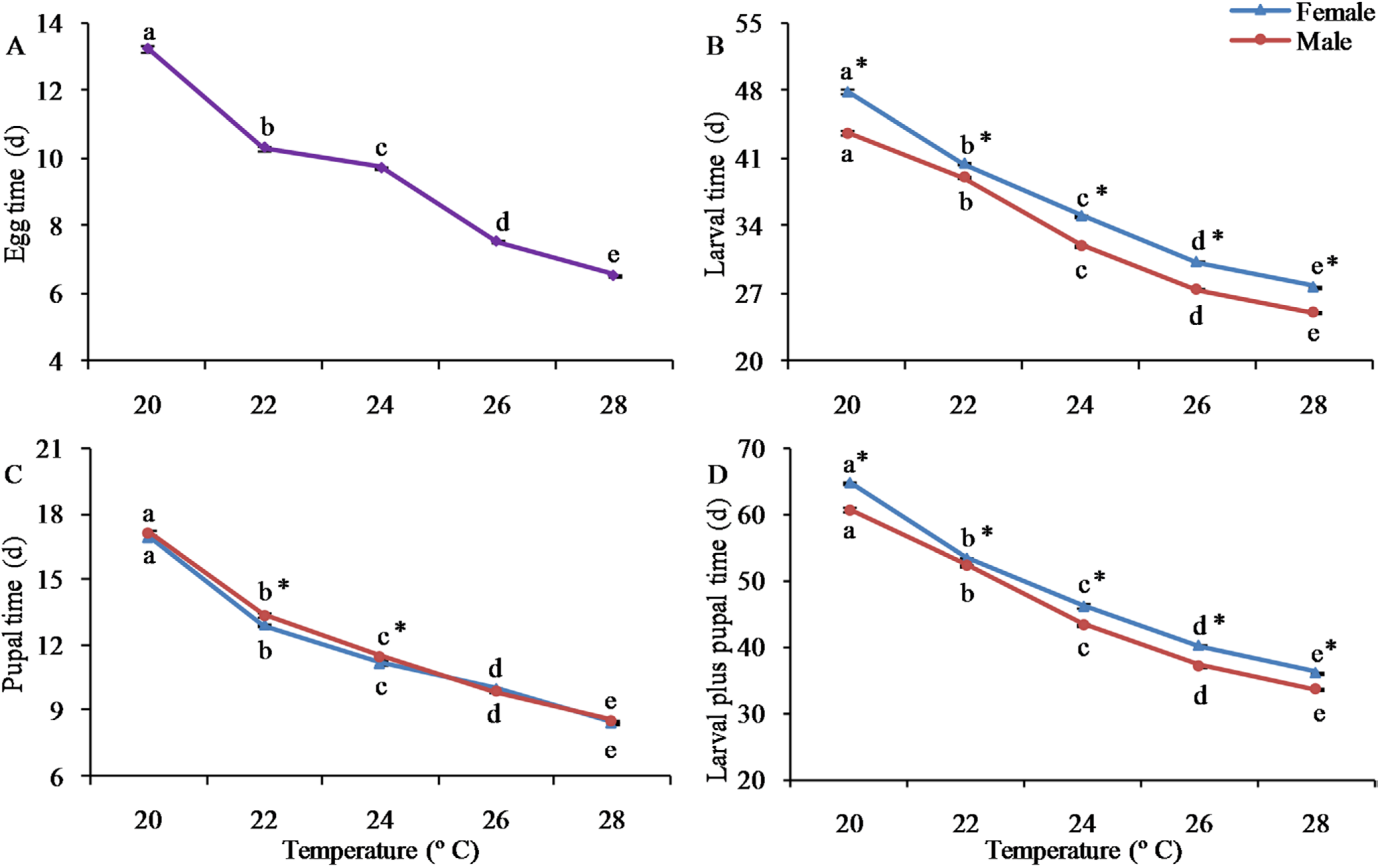
Egg, larval, pupal time and larval plus pupal time of the fall webworm *Hyphantria cunea* at different temperatures. Error bars represent standard errors (SEs). Values with different lowercase letters among populations indicate significant differences at the 0.05 level. The asterisk denotes a significant difference between sexes (*p* < 0.05).

Temperature had a substantial influence on the developmental time from egg to adult stage. The egg incubation period dropped significantly from 13.3 days at 20°C to 6.5 days at 28°C (*F*_4,10_ = 1811.652, *p* < 0.001; Figure 1A). Larval development time was notably influenced by temperature, sex, and their interactions (Table 2). Both female and male larval development times exhibited significant reductions with increasing temperature (Figure 1B), dropping from 47.9 days (females) and 43.7 days (males) days at 20°C to 27.8 days (females) and 25.1 days (males) at 28°C (Table S1). Obvious differences in larval developmental time were detected between the sexes at all temperatures (Figure 1B, Table S1). Pupal time was also likewise significantly impacted by temperature, sex, and their interactions (Table 2). At 20°C, pupal development persisted for 16.9 days in females and 17.1 days in males, while at 28°C, it lasted for 8.5 days in females and 8.6 days in males (Table S1). Notably, significant variations in pupal time were discovered between the sexes at 22°C and 24°C, with males requiring a longer duration than females (Figure 1C, Table S1). The time from hatching to adult emergence was significantly affected by temperature, sex, and their interactions (Table 2). The total development time of females was significantly longer than that of males (Figure 1D). Male adults emerged earlier than females at each temperature: 4 days at 20°C, 1 day at 22°C, 2.8 days at 24°C, 3 days at 26°C, and 2.6 days at 28°C (Figure 1D). These results indicate that this moth exhibits a protandrous emergence pattern. In contrast to pupal development, larval development contributed to protandry in a considerable way.

**TABLE 2:** The results obtained from a linear mixed‐model analysis of fixed effects on larval time, pupal time, time from hatching to adult emergence, pupal weight, growth rate, adult weight, and proportionate weight loss in *Hyphantria cunea* with regard to temperature and sex.

Life‐history traits	Fixed factors	df	*F*‐value	*p*
Larval time	Temperature	4	4304.096	< 0.001
	Sex	1	703.687	< 0.001
	Temperature × sex	4	16.223	< 0.001
Pupal time	Temperature	4	3236.898	< 0.001
	Sex	1	12.605	< 0.001
	Temperature × sex	4	3.530	0.007
Time from hatching to adult emergence	Temperature	4	5746.833	< 0.001
	Sex	1	413.673	< 0.001
	Temperature × sex	4	13.370	< 0.001
Pupal weight	Temperature	4	70.991	< 0.001
	Sex	1	3699.969	< 0.001
	Temperature × sex	4	21.342	< 0.001
Growth rate	Temperature	4	4533.018	< 0.001
	Sex	1	0.039	0.844
	Temperature × sex	4	6.260	< 0.001
Adult weight	Temperature	4	121.240	< 0.001
	Sex	1	11,837.928	< 0.001
	Temperature × sex	4	13.243	< 0.001
Proportionate weight loss	Temperature	4	74.391	< 0.001
	Sex	1	9007.464	< 0.001
	Temperature × sex	4	5.447	< 0.001

### 3.3 Pupal Weight as Well as Growth Rate

**FIGURE 2:**
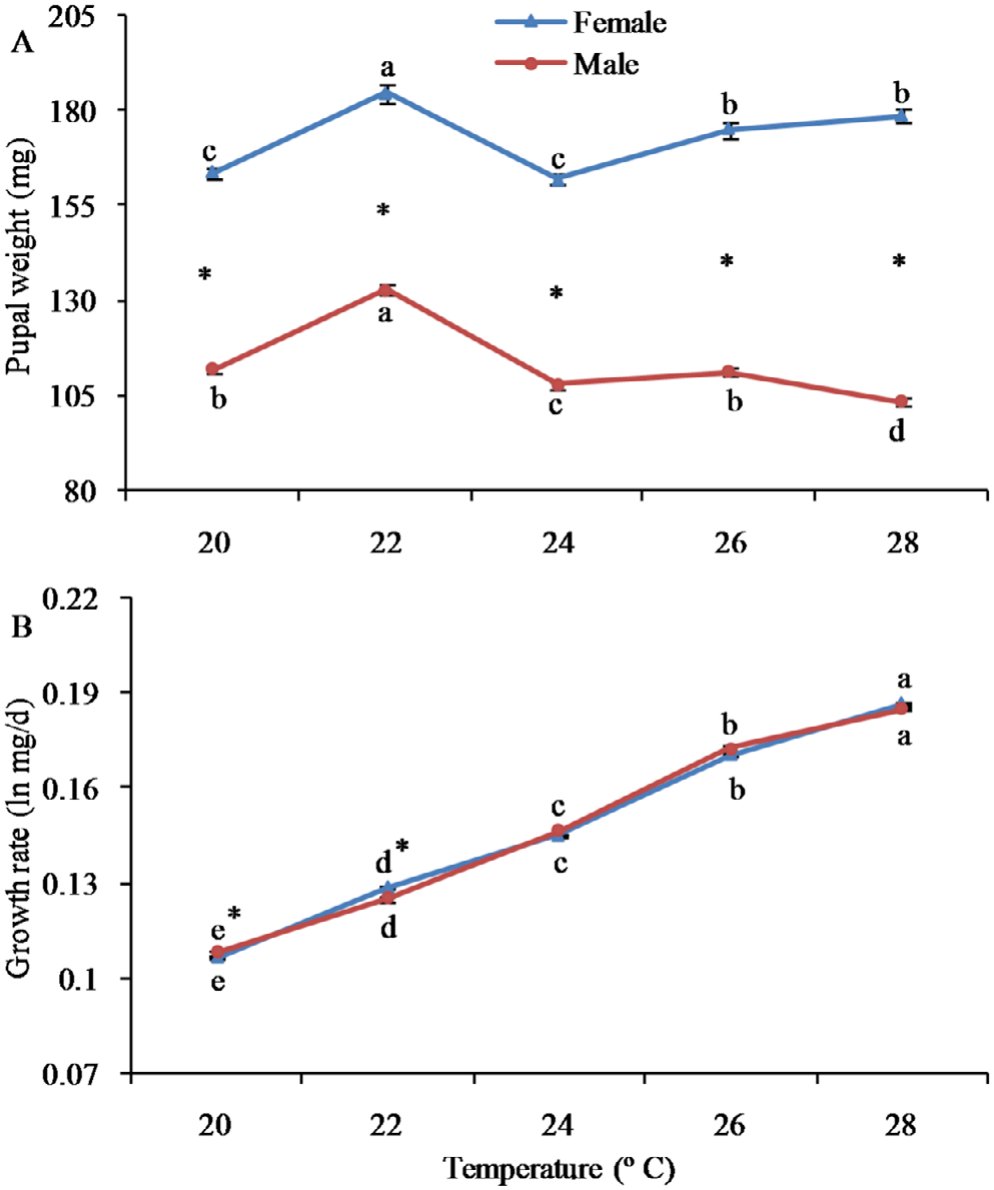
Pupal weight and growth rate of the fall webworm *Hyphantria cunea* at different temperatures. Error bars represent standard errors (SEs). Values with different lowercase letters among populations indicate significant differences at the 0.05 level. The asterisk denotes a significant difference between sexes (*p* < 0.05).

**FIGURE 3:**
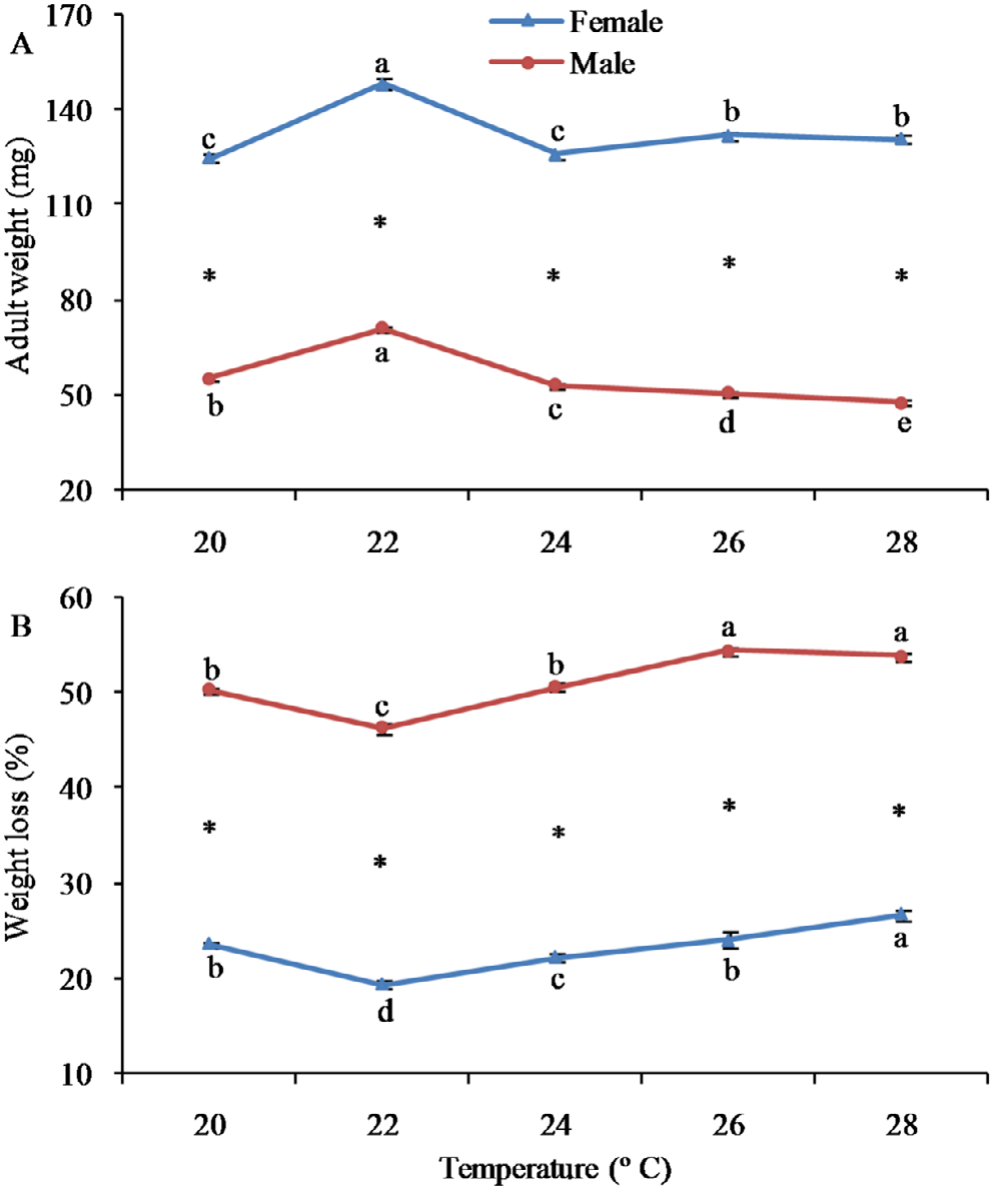
Adult weight and weight loss of the fall webworm *Hyphantria cunea* at different temperatures. Error bars represent standard errors (SEs). Values with different lowercase letters among populations indicate significant differences at the 0.05 level. The asterisk means significant difference between sexes (*p* < 0.05).

Pupal weight was substantially affected by population, sex, and their interactions (Table 2). For females, pupal weight peaked at 22°C (184.6 mg), followed by at 28°C (178.7 mg) and 26°C (175.1 mg), which were significantly higher than 20°C (163.7 mg) and 24°C (162.2 mg). According to the calculation, the weight of female pupae increased by 9.2% at 28°C compared to 20°C. Higher temperatures led to larger female pupae, indicating that this moth does not follow the TSR (a negative connection between size and rearing temperature). For male individuals, the pupal weight reached the highest level at 22°C (133.1 mg), followed by at 20°C (112.1 mg), 26°C (111.3 mg), and 24°C (107.9 mg), all of which were significantly higher than 28°C (103.6 mg) (Figure 3A, see also Table S1). According to the calculation, the weight of male pupae decreased by 8.2% at 28°C compared to 20°C. Elevated temperatures led to smaller male pupae, suggesting that males are particularly sensitive to high temperatures. Additionally, female pupal weight was consistently and significantly higher than that of male pupal weight at all temperatures, indicating a female‐biased SSD. The ratio of female to male body weight was positively correlated with temperature. Specifically, it was noticed that this ratio was 1.38 times at 22°C, 1.5 times at 24°C, 1.57 times at 26°C, and 1.73 times at 28°C (Figure 2A, see also Table S1).

The growth rate was notably influenced by temperature and temperature × sex, yet not by sex. Growth rate rose significantly along with the increase in temperature for both females and males (Figure 2B). At 20°C, the growth rate of males was significantly greater than that of females; however, at 22°C, the growth rate of females was significantly higher than that of males. There were no remarkable differences in the growth rate between the sexes at 24°C, 26°C, and 28°C (Figure 2B).

### 3.4 Adult Weight as Well as Weight Loss

Adult weight was significantly shaped by population, sex, and their interactions (Table 2). The body weight of female adults showed a similar tendency to that of pupae in terms of temperature changes. The highest weight was seen at 22°C, with 28°C and 26°C following (Figure 3A). Females were significantly bigger than males at every temperature.

Weight loss during metamorphosis was significantly shaped by population, sex, and their interactions (Table 2). With the exception of 20°C, weight loss from pupae to adults rose significantly with the increase in rearing temperature (Figure 3B). Male pupae experienced significantly greater loss during metamorphosis compared to female pupae at all temperatures.

### 3.5 Sexual Size Dimorphism

**FIGURE 4:**
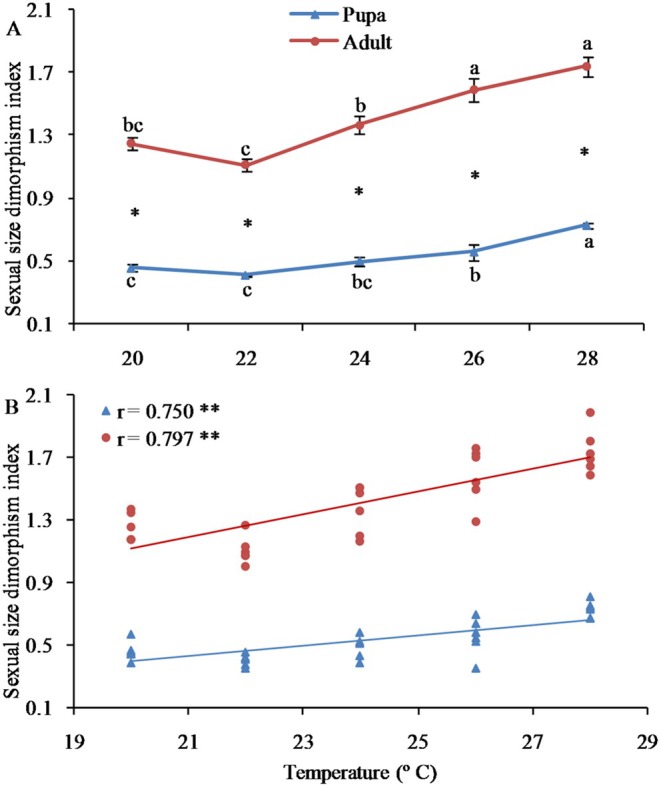
Sexual size dimorphism of the fall webworm *Hyphantria cunea* at different temperatures. Error bars represent standard errors (SEs). Values with different lowercase letters among populations indicate significant differences at the 0.05 level. The asterisk denotes a significant difference between developmental stages (*t*‐test, *p* < 0.05).

Sexual Size Dimorphism (SSD) for both pupae and adults was significantly determined by temperature (Pupae, *F*_4,25_ = 17.923, *p* < 0.001; Adult, *F*_4,25_ = 21.847, *p* < 0.001) and gradually increased with increasing rearing temperature (Figure 4A). The SSD index for pupae was significantly lower than that for adults (all *t‐tests, p* < 0.001). Linear regression analysis disclosed a significant positive correlation between SSD and temperature (Figure 4B).

### 3.6 Biological Parameters of Mated Females

The biological parameters of mated females are presented in Table 3. The mating success rate for 30 adult pairs at temperatures of 20°C, 22°C, 24°C, 26°C, and 28°C was 90%, 70%, 73.3%, 90%, and 76.7%, respectively. No significant differences were detected in the preoviposition period (*F*_4,115_ = 1.032, *p* = 0.394) or in the oviposition period (*F*_4,115_ = 1.408, *p* = 0.236). The lifespan of females was significantly longer at 22°C than at 24°C. Fecundity peaked at 22°C with 806 eggs, followed by 26°C with 739 eggs, 28°C with 730 eggs, and 24°C with 672 eggs; fecundity was significantly lower at 20°C with 607 eggs (*F*_4,115_ = 2.618, *p* = 0.039).

**TABLE 3:** Biological parameters of mated *Hyphantria cunea* females when newly emerged adults reared under different temperatures were transferred to a rearing room (26°C± 2°C, natural photoperiod) for mating and oviposition.

Temperature (°C)	Number of mated females	Preoviposition period (d)	Oviposition period (d)	Longevity of female (d)	Fecundity (eggs/female)
20	27	3.22 ± 0.27 a	2.67 ± 0.21 a	7.78 ± 0.27 ab	606.81 ± 35.58 b
22	21	2.62 ± 0.29 a	2.38 ± 0.26 a	6.90 ± 0.41 b	806.05 ± 63.38 a
24	22	3.23 ± 0.29 a	2.95 ± 0.30 a	7.95 ± 0.12 a	671.91 ± 52.51 ab
26	27	2.81 ± 0.23 a	2.85 ± 0.25 a	7.00 ± 0.32 b	739.11 ± 62.52 ab
28	23	3.04 ± 0.18 a	2.26 ± 0.20 a	7.65 ± 0.25 ab	729.91 ± 40.49 ab

*Note:* Values (±SE) within one column followed by distinct letters are significantly different at the 0.05 level based on one‐way ANOVA and Tukey's HSD multiple tests.

### 3.7 Relationship Between Two Traits

**FIGURE 5:**
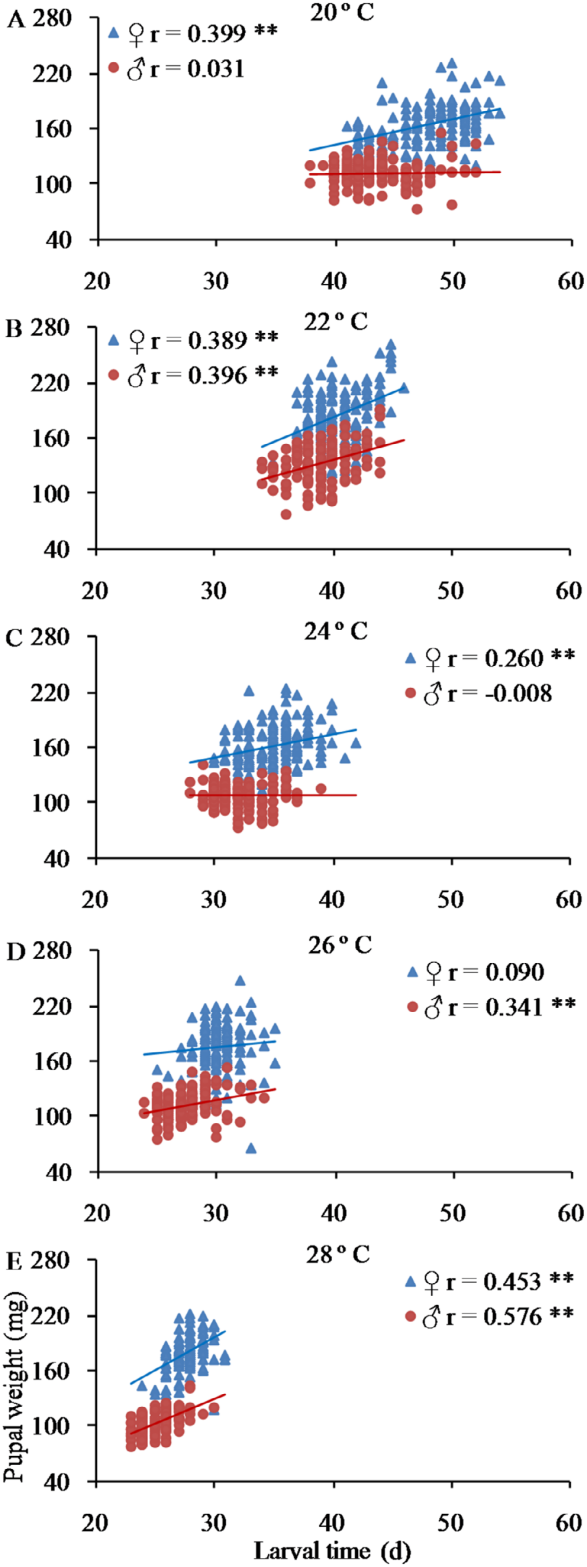
Relationship between larval development time and pupal body weight in the fall webworm *Hyphantria cunea* . The asterisk indicates a significant correlation (**p ≤ 0.01).

**FIGURE 6:**
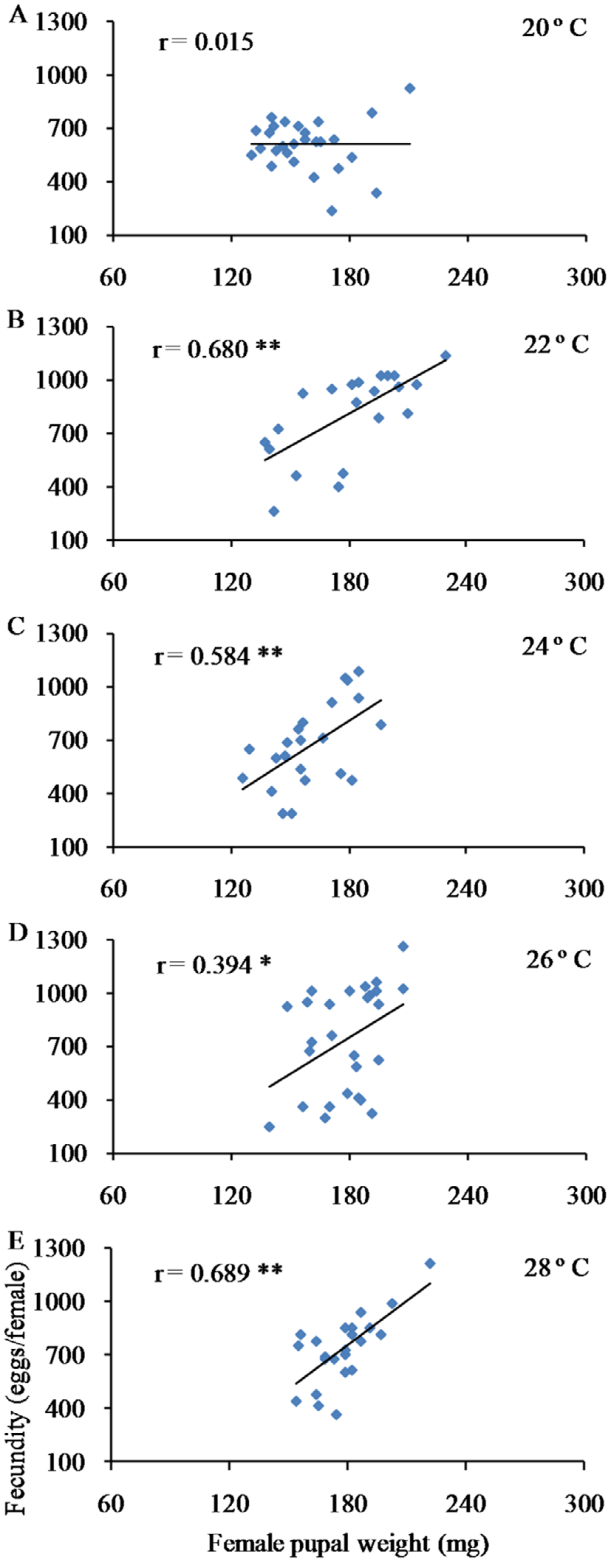
Relationship between female body weight and fecundity in the fall webworm *Hyphantria cunea* . The asterisk indicates a significant correlation (*p ≤ 0.05, **p ≤ 0.01).

As illustrated in Figure 5, an extended larval period was associated with larger pupae. A significant positive correlation was observed between pupa weight and larval development time for females at 20°C, 22°C, 24°C, and 28°C, and for males at 22°C, 26°C, and 28°C (*p* < 0.01). Additionally, heavier female pupae tended to yield a higher number of eggs (Figure 6). At various temperatures, there was a notable positive correlation between female pupa weight and fecundity (*p* < 0.05 or *p* < 0.01), with the exception of 20°C.

## 4 Discussion

Through conducting a meticulous analysis of the life‐history traits of the fall webworm under different temperatures, we have obtained some interesting findings.

We discovered that the period from hatching to adult emergence was significantly longer for females than for males, resulting in early emergence of males (protandry). The protandrous emergence pattern primarily stems from sexual dimorphism in larval development rates rather than pupal duration. It has been demonstrated that protandry occurs in insect species where females mate shortly after emergence and are monogamous throughout their lives (Wiklund and Fagerström 1977; Fagerström and Wiklund 1982). For instance, the protandrous butterfly *Lycaena tityrus* is monandrous and possesses discrete, non‐overlapping generations (Fischer and Fiedler 2000). Protandry in *H. cunea* appears to play a crucial role in reproductive success, given the shorter adult longevity (approximately 7 days) due to degenerated mouthparts and limited time frame (9–58 h post‐emergence) for females to locate males (Yan et al. 2000). Consequently, early male emergence ensures that females are available for mating upon reaching sexual maturity, thereby minimizing potential delays in reproduction.

We found that the TSR did not apply to the female pupal weight in this study. Specifically, the weights of female pupae at 26°C (175.1 mg) and 28°C (178.7 mg) were significantly higher than those at 20°C (163.7 mg) and 24°C (162.2 mg), demonstrating a reverse TSR pattern. This phenomenon is consistent with previous reports by Gomi (2006) and Wang et al. (2024), who observed heavier pupal mass at elevated temperatures in this species. The increased female pupal weight under high temperature indicates that temperature has a stronger effect on the growth rate than the development rate (Von der Have and Jong 1996), where biomass accumulation rate during larval development exhibits higher thermal sensitivity than the developmental progression (Angilletta and Dunham 2003; Zuo et al. 2012). We further observed that male pupal weight decreased significantly under high‐temperature conditions. Specifically, elevated temperatures enhanced female pupal mass (+9.2% at 28°C vs. 20°C) while reducing male mass (−8.2%). Our study is the first to demonstrate that male and female larvae of the fall webworm exhibit differential sensitivity to high temperatures. This sexual dichotomy reflects fundamental differences in resource allocation strategies, with females prioritizing fecundity‐related mass gain and males focusing on optimizing emergence timing. Furthermore, we observed a temperature‐mediated increase in the SSD index, which rose from 1.38 to 1.73 as temperatures increased from 22°C to 28°C, suggesting the influence of climate‐dependent selection pressures. A similar result has been stated in the Asian corn borer *O. furnacalis*, where female pupa weight increased by 12.3%, while male pupal weight decreased by 8.7% at 25°C compared to 20°C (Xia et al. 2019). This temperature‐size response pattern has positive adaptive implications. Under high‐temperature stress, females tend to increase their body size in order to maximize reproductive output, while males reduce their body size to minimize metabolic costs and enhance dispersal efficiency. In addition, based on our field observations in 2024, nearly all larvae hatched after late August were induced into pupal diapause under the conditions of gradually decreasing day length, despite the persistently high temperatures in September (the average daily temperature in September 2024 exceeded 28°C). In a natural setting, this response pattern ensures that individuals entering winter diapause attain relatively higher pupal body weight. As a consequence, females emerging from overwintering pupae tend to produce a greater number of eggs. Our study further offers an additional example showing that reverse TSR in the fall webworm is closely related to diapause characteristics (Fu et al. 2016; Xia et al. 2019). That is, species showing strong photoperiod‐inducing diapause under high temperature conditions may present reverse TSR.

Our study revealed that, across all temperatures, male pupae lost significantly more weight during metamorphosis than females. This finding implies that males either have a greater metabolic rate or are less capable of preventing water loss (Savalli and Fox 1998). Additionally, it was discovered that the weight loss of both sexes rose as the temperature increased, and the degree of SSD also climbed with the growing temperature. Consequently, the SSD of pupae is significantly lower compared to that of adults. These results suggest that sex‐specific weight loss plays a mediating role in SSD in the fall webworm (Testa et al. 2013). According to Rensch's rule, SSD is expected to decline with body size when females are the larger gender (Abouheif and Fairbairn 1997; Fairbairn 1997). However, in our study, the female‐biased SSD in the fall webworm had a tendency to increase with increasing body weight, and the SSD index showed a tendency to rise with increasing temperature. Clearly, these findings do not conform to Rensch's rule (Teder and Tammaru 2005).

We found out that there was a significant positive correlation between pupal weight and larval development time across nearly all tested temperatures, aligning with the theoretical expectation that animals with prolonged growth periods tend to attain larger sizes (Nijhout et al. 2010). Consequently, our findings suggest the existence of a trade‐off between these two traits. The relation between development time and body size has been noted in 77% of Lepidopteran insect species (122 of 146 datasets) (Teder et al. 2014). Nevertheless, a negative correlation between the two traits has been stated in the rice stem borer *C. suppressalis* under field conditions (Huang et al. 2018) and in the Asian corn borer *O. furnacalis* reared under constant temperatures (Xia et al. 2019), where relatively shorter larval developmental times were related to relatively larger pupal weights.

The fecundity advantage hypothesis claims that larger females produce more offspring than smaller females (Honĕk 1993; Andersson 1994; Omkar and Afaq 2013). Our findings are consistent with this hypothesis. In the fall webworm, a significant positive correlation was noticed between female pupal weight and adult fecundity under all temperature conditions. This significant positive connection between pupal weight and adult fecundity has also been documented in other lepidopteran species, like the spruce budworm *Choristoneura fumiferana* (Miller 1957), the sugarcane borer *Diatraea saccharalis* (Bessin and Reagan 1990), the Mexican rice borer *Eoreuma loftini* (Spurgeon et al. 1995), the oriental armyworm *Mythmna separate* (Luo et al. 1995), the moth *Streblote panda* (Calvo and Molina 2005), the diamondback moth *Plutella xylostella* (Zhang et al. 2012), and the fall armyworm *Spodoptera frugiperda* (Huang et al. 2021).

## 5 Conclusions

This study revealed significant sex differences and temperature regulation effects in the life history of fall webworm. The development cycle shortened with increasing temperatures, and males emerged earlier than females, a phenomenon known as protandry. Female pupae exhibited significantly greater body weight at high temperatures, displaying a reverse TSR pattern. In contrast, male pupae showed decreased body weight, indicating greater thermal sensitivity. Additionally, male pupae experienced greater weight loss during metamorphosis compared to females. Elevated temperatures were found to intensify both weight loss and SSD, indicating that sex‐specific weight dynamics play a crucial role in shaping the ontogeny of SSD. Female‐biased SSD increased with rising temperature and body weight, deviating from Rensch's rule. A general trade‐off between larval development time and pupal weight was witnessed, consistent with growth‐allocation theory. Moreover, the strong correlation between female pupal weight and fecundity across temperatures firmly supports the fecundity advantage hypothesis. These findings uncover the mechanism by which metabolic physiology, environmental gradients, and reproductive strategies synergistically shape SSD evolution and life history dynamics.

## Author Contributions

**Hua Lu:** data curation (equal), formal analysis (equal), investigation (equal), methodology (equal), validation (equal), writing – original draft (equal). **Li‐Li Huang:** data curation (equal), funding acquisition (equal), resources (equal), validation (equal), writing – review and editing (equal). **Liang Chen:** investigation (equal), resources (equal). **Sheng‐Bin Wu:** investigation (equal), resources (equal). **Fang‐Sen Xue:** conceptualization (equal), data curation (equal), methodology (equal), project administration (equal), supervision (equal), writing – review and editing (equal). **Xing‐Ping Liu:** conceptualization (equal), data curation (equal), methodology (equal), writing – review and editing (equal). **Hai‐Min He:** conceptualization (equal), data curation (equal), formal analysis (equal), investigation (equal), methodology (equal), software (equal), visualization (equal), writing – review and editing (equal).

## Conflicts of Interest

The authors declare no conflicts of interest.

## Supporting information


**TABLE S1:** Life‐history data (mean ± SE) for female and male of *Hyphantria cunea* at 20°C, 22°C, 24°C, 26°C, and 28°C.

## Data Availability

Empirical data have been archived in Data Dryad: https://doi.org/10.5061/dryad.5qfttdzkk.
