# Supplementary material for: Life‐History Responses of the Fall Webworm Hyphantria cunea to Temperature Change: Not Following the Temperature–Size Rule
Source: Ecol Evol. 2025 Oct 1;15(10):e72225. doi: 10.1002/ece3.72225 (PMC12488214; doi:10.1002/ece3.72225)
Supplement: Supplementary file 1 — TABLE S1: Life‐history data (mean ± SE) for female and male of Hyphantria cunea at 20°C, 22°C, 24°C, 26°C, and 28°C. [file ECE3-15-e72225-s001.doc]

**S1 TABLE** | Life-history data (mean ± SE) for female and male of *Hyphantria cunea* at 20, 22, 24, 26 and 28 °C.

| **Life-history traits** | **20 °C** | | **22 °C** | | **24 °C** | | **26 °C** | | **28 °C** | |
| --- | --- | --- | --- | --- | --- | --- | --- | --- | --- | --- |
|  | **Female** | **Male** | **Female** | **Male** | **Female** | **Male** | **Female** | **Male** | **Female** | **Male** |
|  | N = 163 | N = 159 | N = 163 | N = 149 | N = 170 | N = 164 | N = 167 | N = 171 | N = 138 | N = 174 |
| Larval time (d) | 47.87 ± 0.25 a * | 43.67 ± 0.23 a | 40.49 ± 0.16 b * | 39.04 ± 0.19 b | 35.09 ± 0.18 c * | 32.02 ± 0.16 c | 30.25 ± 0.12 d * | 27.36 ± 0.14 d | 27.77 ± 0.11 e * | 25.06 ± 0.10 e |
| Pupal time (d) | 16.93 ± 0.12 a | 17.13 ± 0.11 a | 12.94 ± 0.08 b * | 13.41 ± 0.07 b | 11.20 ± 0.08 c * | 11.48 ± 0.08 c | 10.02 ± 0.08 d | 9.90 ± 0.07 d | 8.46 ± 0.06 e | 8.56 ± 0.05 e |
| Time from hatching to adult emergence (d) | 64.80 ± 0.31 a * | 60.81 ± 0.29 a | 53.44 ± 0.20 b * | 52.45 ± 0.22 b | 46.29 ± 0.21 c * | 43.49 ± 0.20 c | 40.27 ± 0.16 d * | 37.26 ± 0.17 d | 36.23 ± 0.12 e * | 33.62 ± 0.11 e |
| Pupal weight (mg) | 163.73 ± 1.68 c * | 112.10 ± 1.06 b | 184.61 ± 2.18 a * | 133.10 ± 1.68 a | 162.20 ± 1.72 c * | 107.93 ± 0.94 c | 175.14 ± 1.93 b * | 111.25 ± 1.06 b | 178.70 ± 1.69 b * | 103.55 ± 0.92 d |
| Growth rate (ln mg/d) | 0.1067 ± 0.0005 e * | 0.1083 ± 0.0006 e | 0.1288 ± 0.0005 d * | 0.1253 ± 0.0006 d | 0.1453 ± 0.0007 c | 0.1466 ± 0.0008 c | 0.1708 ± 0.0008 b | 0.1726 ± 0.0008 b | 0.1868 ± 0.0006 a | 0.1852 ± 0.0006 a |
| Adult weight (mg) | 124.81 ± 1.26 c * | 55.54 ± 0.55 b | 148.35 ± 1.65 a * | 71.06 ± 1.03 a | 126.01 ± 1.40 c * | 53.16 ± 0.58 c | 131.96 ± 1.33 b * | 50.70± 0.66 d | 130.86 ± 1.45 b * | 47.76 ± 0.60 e |
| Weight loss (%) | 23.66 ± 0.29 b * | 50.28 ± 0.37 b | 19.41 ± 0.33 d * | 46.40 ± 0.55 c | 22.27 ± 0.37 c * | 50.63 ± 0.42 b | 24.08 ± 0.79 b * | 54.41 ± 0.43 a | 26.74 ± 0.48 a * | 53.88 ± 0.41 a |

Note: Values within one row followed by different letters are significantly different at 0.05 level based on one-way ANOVA and Tukey’s HSD multiple tests. * means significantly different between sexes (*p* < 0.05)
